# Advances in Bio-Based Materials for Food Packaging Applications

**DOI:** 10.3390/membranes12080735

**Published:** 2022-07-27

**Authors:** Fabrice Gouanvé

**Affiliations:** Univ Lyon, CNRS, UMR 5223, Ingénierie des Matériaux Polymères, Université Claude Bernard Lyon 1, INSALyon, Université Jean Monnet, CEDEX, F-69622 Villeurbanne, France; fabrice.gouanve@univ-lyon1.fr

Food packaging is defined as a group of boxes, envelopes, papers, and coatings that are employed in order to increase the shelf life of foodstuffs. This means that any food packaging system aims to avoid or slow down bacterial contamination and deterioration and to ensure that properties such as firmness, color, and flavor are maintained for as long as possible. Nowadays, in addition to the traditional concept of packaging as an impenetrable barrier, novel systems are required, such as modified atmosphere packaging and active packaging. Polymeric membranes, thanks to their unique properties of permselectivity and the ability to sustain controlled delivery, can be successfully used for food packaging.

Currently, the packaging industry relies heavily on the use of non-biodegradable, petroleum-derived polymer materials, which raises some concerns from environmental perspectives. Furthermore, because of their lack of biodegradability, non-biodegradable, petroleum-based membranes can pose significant waste disposal problems. So, the development of high-performance bio-based materials is an important factor in the sustainable growth of the packaging industry.

This Special Issue entitled “Advances on Bio-Based Materials for Food Packaging Applications” in the journal *Membranes* aims to assess recent developments regarding bio-based polymers for packaging, their evaluation of sustainability, and their end-of-life options. Various polymers are mentioned, including Polybutylene Succinate, Polylactic acid, Poly(3-hydroxybutyrate-co-3-hydroxyvalerate), Starch, Arabinoxylan, Agar, Mung bean flour, Chitosan, Pectin, etc. There are 16 contributions, namely, 2 reviews and 14 research articles, in this Special Issue.

Salgado-Cruz et al. [1] performed a systematic literature review focused on the scientific production, trends, and characteristics of a knowledge domain of high worldwide importance, namely, the use of chitosan as a coating for postharvest disease biocontrol in fruits and vegetables, which are generated mainly by fungi and bacteria such as *Aspergillus niger*, *Rhizopus stolonifera*, and *Botrytis cinerea*. For this, an analysis of 875 published documents in the Scopus database was performed for the years 2011 to 2021. The information of the keywords’ co-occurrence was visualized and studied using the free access VOSviewer software to show the trend of the topic in general. The study showed an increase in the research of the chitosan and nanoparticle coating applications to decrease postharvest damage caused by microorganisms (e.g., fungi and bacteria), as well as to improveme the shelf life and quality of the products. Finally, this work can provide a useful perspective for future research in the studied field since it demonstrates the existence of an emerging area of study that is intended to reduce a global problem caused by the generation of agro-industrial waste due to the loss of post-harvest damaged crops.

Ma et al. [2] reviewed the upcoming progress in the classification, preparation, and application of food packaging indicators. Equally, the feasibility of 3D printing in the preparation of intelligent food packaging indicators is also discussed in detail, as well as the limitations and future directions of smart food packaging. Intelligent packaging can feel, inspect, and record external or internal changes in food products to provide further information about food quality. As widely used intelligent food packaging, indicators are mainly applied to predict the shelf life of food products and to communicate food quality and safety information, as well as other characteristics, to consumers by the presence of certain chemical or biological substances inside the food packaging. Importantly, intelligent packaging indicators will account for a significant proportion of the food industry’s production, with promising application potential.

Cosquer et al. [3] prepared Polybutylene Succinate (PBS)/Graphene nanoplatelets (GnP) nanocomposites over a range of GnP from 0 to 1.35 wt%. by a melt process. A mixture of individual graphene nanosheets and aggregates was obtained by the addition of GnP in the PBS matrix. The presence of these fillers did not significantly modify the morphology, the crystalline microstructure of the matrix, or its thermal stability. However, a slight reinforcement effect of PBS was reported in the presence of GnP. The water sorption isotherm modelling with the Guggenheim, Andersen, and De Boer (GAB) equation and the Zimm–Lundberg theory allowed a phenomenological analysis at the molecular scale. The presence of GnP did not modify the water sorption capacity of the PBS matrix. From a kinetic point of view, a decrease in the diffusion coefficient with the increasing GnP content was obtained and was attributed to a tortuosity effect. The influence of water activity was discussed over a range of 0.5 to 1 and 0 to 0.9 for water and dioxygen permeability. Improvements in the barrier properties by 38% and 35% for water and dioxygen permeability, respectively, were obtained.

Doineau et al. [4] studied the structural and physical-chemical stability of neat poly(3-hydroxybutyrate-co-3-hydroxyvalerate) (PHBV) and PHBV/(ligno-)cellulosic fiber-based biocomposite materials under dishwashing conditions in order to consider a possible reuse of these types of materials as food contact materials. This work therefore highlights the impact of successive dishwashing cycles on the physical-chemical and structural stability of such materials. Several parameters were considered to assess this stability, such as the visual aspect and color, the microstructure, the thermal and tensile properties, and the overall migration in food liquid simulants. The effects of fiber composition, morphology, and content were investigated by selecting three types of commercial (ligno-)cellulosic fibers and two filler contents (20 and 40 wt%). A great potential for the reuse of PHBV films was highlighted by their high stability after up to at least 50 dishwashing cycles. However, the addition of (ligno-)cellulosic fillers negatively impacts the stability of PHBV-based materials, especially due to the hygroscopic behavior of (ligno-)cellulosic fillers and the heterogenous microstructure of biocomposites, with at best up to 10 possible dishwashing cycles for ultra-pure cellulose. In conclusion, reuse, including dishwashing steps, can be considered for neat PHBV materials, while this should be prohibited for PHBV/(ligno-)cellulosic-fiber-based biocomposite materials.

Chang et al. [5] investigated the increase in antibacterial activity of chitosan–polylactic acid (PLA) composite film by adding nisin and ethylenediaminetetraacetic acid (EDTA). They evaluated the mechanical, physicochemical, and antibacterial properties of various PLA composite films, as well as the enhancement effect of PLA composite films with EDTA + nisin on the preservation of grouper fillets. Films of PLA alone, PLA plus chitosan, PLA plus nisin + EDTA, and PLA plus chitosan plus nisin + EDTA were prepared. The addition of nisin and EDTA to the chitosan–PLA matrix significantly increased the antibacterial activity of the chitosan–PLA film and did not significantly affect the mechanical strength of the chitosan–PLA film. Covering fish fillets with the nisin + EDTA-added chitosan–PLA composite film effectively reduced the mesophile, coliform, and spoilage bacteria counts, as well as the TVBN content during storage at 25 °C and 4 °C. Therefore, adding EDTA and nisin to the chitosan–PLA film is a promising solution to meet consumers’ demand for natural preservatives and may be a breakthrough technology for preserving fresh food and extending shelf life.

Weng et al. [6] investigated the elaboration of biodegradable films by casting process from a purified arabinoxylan extract from corn fiber. The decolorization of a purified arabinoxylan extract from corn fiber was achieved using hydrogen peroxide as a decolorizing agent. Decolorized films of arabinoxylan were prepared with glycerol as a plasticizer and citric acid as a cross-linker. Though the cross-linking reaction was not successful, since the film incorporated with citric acid still presented a high solubility in water, the films showed promising properties. Decolorized films with glycerol and citric acid retained a significant antioxidant activity, and values of water vapor permeability were similar to those of non-decolorized ones and other polysaccharides. In addition, the decolorization process did not substantially affect the films’ mechanical properties under perforation tests. New strategies are necessary to improve the conditions for cross-linking reactions, either by changing the solvent or choosing a different cross-linker. To improve the properties of resulting films, arabinoxylan blends with other polysaccharides or biopolymers with hydrophobic nature are envisaged.

Lai et al. [7] studied the impact of botanical sources (including water chestnuts, maize and potatoes) on film performance of the different starch-based packaging films. All tested films were optically transparent in the visible range (400–700 nm), with their percentage of transmittance ranging from 50% to 80%. Variations in the botanical sources of starch have no significant impact on the color parameters (including L*, a*, and b*) and morphological features of the films but affect the water vapor permeability, maximum tensile strength, and elongation at break. Starch films from water chestnut show the highest percentage of transmittance, whereas those from potatoes are the opaquest. No observable change in the intensity of clusteroluminescence occurs when a packaging bag generated from starch is used to package fresh or frozen chicken breast meat; however, a remarkable decline in the intensity of luminescence is noted when the frozen meat is thawed inside the bag. The obtained results reveal the impact of starch sources on the performance of starch films in food packaging and demonstrate the possibility of using the clusteroluminescence of starch as an indicator to reveal the state of packaged frozen food.

Hong et al. [8] investigated the changes in microbiological and physicochemical properties of fresh beef loin packaged with the agar/silver nanoparticles (AgNP) composite films using conventional air-sealed packaging during storage at refrigerated temperature. In this work, raw beef cuts were directly inoculated with *Listeria monocytogenes* and *Escherichia coli* O157:H7 and stored in the air-sealed packages combined with the agar films at 5 °C for 15 days. No significant antimicrobial effect was found against the mesophilic aerobes, lactic acid bacteria, and coliform bacteria. However, the composite films could partly prevent beef samples from directly contacting oxygen, maintaining the meat color, and retarding oxidative rancidity. Experimental results suggested that the AgNP-incorporated agar films can potentially be applied in packaged raw meats as an active food packaging material to inhibit microbial and physicochemical quality deterioration during distribution and sale. Further research is needed to improve the effectiveness of sealed packaging with the agar/AgNP composite films. Combining antimicrobial packaging with modified atmosphere conditions can provide a much more successful way to maintain the quality attributes of fresh red meats and secure food safety during storage. For the practical use of AgNP-containing films in food packaging applications, however, assessing the real amount of silver in contact with food is required to verify the fulfillment of the EU and other regulations. In this aspect, more research should be carried out on the migration of AgNPs from the packaging to the food matrix and their cytotoxic effects on human health.

Keawpeng et al. [9] studied the elaboration of films made from mung bean flour incorporated with longkong pericarp extract (LPE). Longkong (*Aglaia dookkoo Griff*.) fruit is a non-climacteric tropical fruit native to Thailand. Longkong contains three parts: pericarp, flesh, and seeds, and among them, the pericarp is the major part, which contains an abundant level of polyphenolics. The films were elaborated using an emulsion process using ultrasonication at different time durations. Then, the edible phyto-film was cast and checked for various physicochemical and functional properties. Sonication at different time periods positively influenced the phytofilm and improved various physicochemical, antioxidant, and antimicrobial activities. The addition of LPE increased the antimicrobial and antioxidant activities of the film. The phyto-film emulsion, when sonicated at different times, helped the immobilization of the LPE in the film pores and thus promoted the functional property of the film. Sonication treatment at a prolonged period caused the thickness, solubility, and water vapor permeability of the phyto-film to be decreased. The sonicated phyto-film emulsion heavily decreased its droplet size below 200 nm and changed into a nano-emulsion. The prolonged duration of sonication maintained the zeta potential value below −30 mV, making it a strong and stable film. The prolonged sonication effectively increased the antioxidant and antimicrobial functionality of the film and made it a suitable candidate for applications in the food and drug industries. The present study recommends that phyto-film emulsion sonicated for 6–8 min before casting it into film could be an excellent processing technique that widely improves the film’s functionality in a variety of ways.

Keawpeng et al. [10] investigated the effects of sonication and clove oil incorporation on the improvement of physical, antioxidant, and antimicrobial properties and the lipid oxidation inhibiting abilities of mung bean flour (MF)-based films. There were three groups of films tested: (1) MF: mung bean flour alone; (2) MFC: MF incorporated with 2% clove oil (C); and (3) MFCU: MFC prepared with sonication (25 kHz, 100% amplitude, 10 min). The film thickness and bulk density showed slight differences, and moisture content, solubility, and water vapor permeability significantly differed between the formulations. Tensile strength, elongation at break, and Young’s modulus were highest for the MFCU films, followed by MFC and MF in rank order. Furthermore, the Fourier-transform infrared spectroscopy results also demonstrated that the clove oil and sonication treatment improved the interconnections of the biopolymers, thus increasing the physical strength of the film. Phytochemicals in terms of total phenolics and total flavonoids were elevated in the MFCU films and contributed to stronger radical scavenging abilities (*p* < 0.05). MFC and MFCU films showed a strong antibacterial control of the Gram-positive *Staphylococcus aureus* (*S. aureus*) and also of the Gram-negative *Campylobacter jejuni* (*C. jejuni*). Overall, the lipid oxidation indicators Thiobarbituric acid reactive substances (TBARS, peroxide value, p-anisidine value, and totox value) showed significantly high inhibition, attributed to radical scavenging activities in the MFCU and MFC samples. The mung bean flour films incorporated with clove oil and prepared with sonication have good potential as packaging materials for food due to strong physical, antimicrobial, and antioxidant properties, as well as lipid oxidation inhibiting abilities.

Meng et al. [11] fabricated pH-sensing films employing hydroxypropyl guar (HPG), 1-butyl-3-methylimidazolium chloride (BmimCl), and anthocyanin (Anth). (BmimCl) was used as a plasticizer and tuner, and Anth was used as a pH-sensitive dye. In addition, the effects of adding cellulose nanocrystals (CNC) into the composite films upon the films’ structures and physicochemical properties were elucidated. The addition of CNC promoted more compact film structures. For instance, the HPG/CNC/IL/Anth and CNC/IL/Anth films exhibited dense and compact structures. They also showed excellent performance for pH detection applications, including easy identification, high sensitivity, and fast color responses (6 and 2 s) to pH changes. Moreover, the films exhibited good mechanical properties, good oxygen and water vapor barrier properties, excellent solvent resistance properties, high durability (>6 months), and low-temperature (<50 °C) resistance. These results expand the application range of pH-sensing films containing CNC in the fields of food freshness detection and intelligent packaging.

Jancikoca et al. [12] prepared edible films based on carrageenan/chitosan and incorporated them into the following matrices: the natural extracts of Clitoria ternatea, Brassica oleracea, and Ipomea batatas. The films were characterized by TPC (total polyphenols content), antioxidant activity, and textural properties. Experimentally produced films were added in the packaging of freshly cut apple pieces, and the apple pieces were dipped into the films produced from carrageenan and chitosan. The appearance of the samples was monitored, as were the antioxidant activity and the total polyphenol content. The intelligent properties of films were evaluated, too. The properties of experimentally prepared edible films showed that the total polyphenol content increased with the addition of extracts, but there were significant differences not only caused by the presence of natural extracts in edible films but also by the polymer used as the basic matrix—κ-carrageenan or chitosan. Another finding that can be emphasized is that the strength of the κ-carrageenan films was not significantly affected by the addition of extracts. The κ-carrageenan films were stronger than chitosan films. The intelligent properties of chitosan films were most visible with the addition of red cabbage extract; in the case of blue tea and sweet potato extract additions, the differences were not visible. The packaging of fresh-cut apple pieces showed different behavior in the samples; when the apple pieces were coated in chitosan, their color was browner than in that of κ-carrageenan coating. In most cases, the carrageenan coating samples showed better antioxidant properties and greater total polyphenol content than that of apple samples immersed in chitosan coating.

Chalapud et al. [13] elaborated Pectin films with sunflower wax addition produced by electrospraying. Solutions and emulsions were prepared and characterized. Film-forming solutions and wax-added electrosprayed films were physical and structurally evaluated. The addition of sunflower wax to the film-forming solutions reduces conductivity while raising surface tension and density, whereas the type of pectin had a larger impact on viscosity, with the low-methoxyl solution having the highest value. These changes in physical solution properties influenced the film characteristics, observing thicker films with lower water vapor transmission rate (WVTR) when adding wax. Micrographs obtained by scanning electron microscopy (SEM) revealed the presence of wax particles as small spherical shapes, having a good distribution through the sectional area of films. According to X-ray diffraction (XRD), atomic force microscopy (AFM), and mechanical properties analyses, the presence of wax had an impact on the degree of crystallinity, producing a more amorphous and rougher film’s structure, without affecting the elongation percentage and the tensile stress (*p* > 0.05). Despite the presence of voids, and the more crystalline structure in LM films, the addition of sunflower waxes to pectin films resulted in a decrease in molecular mobility and water molecule accessibility and therefore reduced the rate of water vapor transmission, demonstrating that waxes are an effective water vapor flux barrier and that the formed network had good water resistance. These results showed that wax addition improves the physical properties of films, while the suitability of using both pectins and the electrospraying technique was demonstrated.

Préfol et al. [14] prepared thin transparent breathable films for food packaging applications by the solvent casting method from both the binary blends Pebax^®^ MH1657 copolymer/hydroxyl-terminated polyethylene glycol (PEGOH) and Pebax^®^ MH1657/polyethylene glycol dimethyl ether (PEGDME), as well as the ternary blend Pebax^®^ MH1657/PEGOH/PEGDME with a 50/50 and 37.5/62.5 PEGOH/PEGDME weight ratio for additive amounts between 0 and 50 wt%. The microstructures of these materials were investigated by differential scanning calorimetry (DSC) and wide-angle X-ray scattering (WAXS) analyses. Regardless of the PEG’s nature, for a PEG amount inferior to 30 wt%, the Pebax^®^ and PEG phases were totally miscible. For higher amounts, a phase separation was obtained. In the presence of PEG, a decrease in crystallinity was obtained. The effects of the nature and amount of PEG on the thermo-mechanical, hydration, and gas (CO_2_, O_2_) transport properties were investigated. A study of the film’s stability in terms of composition over time was also performed. From this work, a wide range of films could be proposed with a stable composition over time and adjustable mechanical and gas transport properties for the prolongation of the shelf-life of highly breathable fresh products.

Danh et al. [15] investigated the use of essential oils (EOs) as the safe alternative to control anthracnose disease on mango fruits of Cat Hoa Loc variety caused by *Colletotrichum* sp. The pathogen was isolated from the infected Cat Hoa Loc mangoes and identified by morphology and DNA sequencing of the ITS region. Six EOs (cinnamon, basil, lemongrass, peppermint, coriander, and orange) were chemically analyzed by GC–MS. The antifungal activities of the EOs were studied in vitro and in vivo. The results showed that the isolated pathogen was *Colletotrichum acutatum*. Cinnamon, basil, and lemongrass EOs effectively inhibited the growth of *C. acutatum* in descending order of cinnamon, basil, and lemongrass. However, with the exception of basil oil, they all severely damaged fruit peels. The antifungal activity was closely related to the main compounds of EOs. Basil EOs effectively controlled anthracnose development on Cat Hoa Loc mangoes artificially infected with C. acutatum, and its effectiveness was comparable to that of fungicide treatment. Consequently, basil EOs can be used as a biocide to control anthracnose on post-harvest Cat Hoa Loc mangoes.

Arboleda Mejia et al. [16] investigated the effects of the microfiltration process (MF) on the physicochemical composition of orange prickly pear juice. Specifically, the effects of the process on the functional properties of the fruit were examined. In addition, different parameters of the microfiltration process (e.g., limiting transmembrane pressure (TMP_lim_) and rejection of compounds) were investigated. The viability of the processing terms of productivity (permeate flux of 77.80 L/h) and the rejection of selected membranes towards specific compounds was analyzed. The quality of the clarified juice was also analyzed for total antioxidants (TEAC), betalains content (mg/100 g wet base), turbidity (NTU), and colorimetry parameters (L, a*, b*, Croma and H). The MF process permitted an excellent level of clarification, reducing the suspended solids and turbidity of the fresh juice. In the clarified juice, a decrease in total antioxidants (2.03 TEAC) and betalains content (4.54 mg/100 g wet basis) was observed as compared to the fresh juice. This could be explained by the degradation of bioactive compounds that provide antioxidant activity due to different factors, such as exposure to oxygen and light. Furthermore, there were significant changes in color properties due to the effects of the L, a*, b*, C, and h values after the removal of the turbidity of the juice. The turbidity also decreased (from 164.33 to 0.37 NTU). Despite the decrease in pigment in the juice, the process of membrane microfiltration is recommended due to the gentle treatment during separation, the reduced damage to the product (due to the lack of heat treatments), the reduction in energy consumption, and the lower equipment costs. The important properties observed, the desirable change in color, and the decrease in turbidity all represent possible benefits for commercial production.

In conclusion, the findings and critical discussions from these contributions highlight the importance of the use of bio-based polymers to replace petroleum-derived polymer materials for food packaging applications. Numerous materials have demonstrated their performance to increase the shelf life of foodstuffs. So, the development of high-performance bio-based materials is an important factor in the sustainable growth of the packaging industry. This Special Issue introduces guidelines concerning the recent developments regarding bio-based polymers for packaging applications.

## References

[1] Salgado-Cruz M.d.l.P., Salgado-Cruz J., García-Hernández A.B., Calderón-Domínguez G., Gómez-Viquez H., Oliver-Espinoza R., Fernández-Martínez M.C., Yáñez-Fernández J. (2021). Chitosan as a Coating for Biocontrol in Postharvest Products: A Bibliometric Review. Membranes.

[2] Ma Y., Yang W., Xia Y., Xue W., Wu H., Li Z., Zhang F., Qiu B., Fu C. (2022). Properties and Applications of Intelligent Packaging Indicators for Food Spoilage. Membranes.

[3] Cosquer R., Pruvost S., Gouanvé F. (2021). Improvement of Barrier Properties of Biodegradable Polybutylene Succinate/Graphene Nanoplatelets Nanocomposites Prepared by Melt Process. Membranes.

[4] Doineau E., Rol F., Gontard N., Angellier-Coussy H. (2022). Physical-Chemical and Structural Stability of Poly(3HB-Co-3HV)/(Ligno-)Cellulosic Fibre-Based Biocomposites over Successive Dishwashing Cycles. Membranes.

[5] Chang S.-H., Chen Y.-J., Tseng H.-J., Hsiao H.-I., Chai H.-J., Shang K.-C., Pan C.-L., Tsai G.-J. (2021). Applications of Nisin and EDTA in Food Packaging for Improving Fabricated Chitosan-Polylactate Plastic Film Performance and Fish Fillet Preservation. Membranes.

[6] Weng V., Brazinha C., Coelhoso I.M., Alves V.D. (2021). Decolorization of a Corn Fiber Arabinoxylan Extract and Formulation of Biodegradable Films for Food Packaging. Membranes.

[7] Lai W.-F., Wong W.-T. (2022). Edible Clusteroluminogenic Films Obtained from Starch of Different Botanical Origins for Food Packaging and Quality Management of Frozen Foods. Membranes.

[8] Hong S.-I., Cho Y., Rhim J.-W. (2021). Effect of Agar/AgNP Composite Film Packaging on Refrigerated Beef Loin Quality. Membranes.

[9] Keawpeng I., Paulraj B., Venkatachalam K. (2022). Antioxidant and Antimicrobial Properties of Mung Bean Phyto-Film Combined with Longkong Pericarp Extract and Sonication. Membranes.

[10] Keawpeng I., Lekjing S., Paulraj B., Venkatachalam K. (2022). Application of Clove Oil and Sonication Process on the Influence of the Functional Properties of Mung Bean Flour-Based Edible Film. Membranes.

[11] Meng Y., Cao Y., Xiong K., Ma L., Zhu W., Long Z., Dong C. (2021). Effect of Cellulose Nanocrystal Addition on the Physicochemical Properties of Hydroxypropyl Guar-Based Intelligent Films. Membranes.

[12] Jancikova S., Dordevic D., Tesikova K., Antonic B., Tremlova B. (2021). Active Edible Films Fortified with Natural Extracts: Case Study with Fresh-Cut Apple Pieces. Membranes.

[13] Chalapud M.C., Baümler E.R., Carelli A.A., Salgado-Cruz M., Morales-Sánchez E., Rentería-Ortega M., Calderón-Domínguez G. (2022). Pectin Films with Recovered Sunflower Waxes Produced by Electrospraying. Membranes.

[14] Préfol T., Gain O., Sudre G., Gouanvé F., Espuche E. (2021). Development of Breathable Pebax®/PEG Films for Optimization of the Shelf-Life of Fresh Agri-Food Products. Membranes.

[15] Danh L.T., Giao B.T., Duong C.T., Nga N.T.T., Tien D.T.K., Tuan N.T., Huong B.T.C., Nhan T.C., Trang D.T.X. (2021). Use of Essential Oils for the Control of Anthracnose Disease Caused by Colletotrichum Acutatum on Post-Harvest Mangoes of Cat Hoa Loc Variety. Membranes.

[16] Mejia J.A.A., Yáñez-Fernandez J. (2021). Clarification Processes of Orange Prickly Pear Juice (*Opuntia* Spp.) by Microfiltration. Membranes.

